# The Role of Near-Infrared Fluorescence with Indocyanine Green in Robot-Assisted Partial Nephrectomy: Results from an Updated Systematic Review and Meta-Analyses of Controlled Studies

**DOI:** 10.3390/medicina61101735

**Published:** 2025-09-24

**Authors:** Andrea Panunzio, Rossella Orlando, Federico Greco, Clara Cerrato, Serena Domenica D’Elia, Laura Marinaci, Federica Manno, Aliasger Shakir, Michele Battaglia, Willy Baccaglini, Antonio Benito Porcaro, Alessandro Antonelli, Andre Abreu, Alessandro Tafuri

**Affiliations:** 1 Dott. Alessandro Tafuri Medical Center, 73100 Lecce, Italy; andrea.panunzio@asl.lecce.it; 2 Department of Urology, Vito Fazzi Hospital, 73100 Lecce, Italy; rossella.orlando@asl.lecce.it (R.O.); serenadomenica.delia@asl.lecce.it (S.D.D.); laura.marinaci@asl.lecce.it (L.M.); federica.manno@asl.lecce.it (F.M.); 3 Ultrasound Radiogenomics AI Center, 70726 San Pancrazio Salentino, Italy; federico.greco@unicampus.it; 4 Department of Radiology, Cittadella della Salute, Azienda Sanitaria Locale di Lecce, 73100 Lecce, Italy; 5 Research Unit of Radiology, Department of Medicine and Surgery, Università Campus Biomedico di Roma, 00128 Roma, Italy; 6 Department of Urology, University Hospital Southampton NHS Trust, Southampton SO16 6YD, UK; clara.cerrato@nhs.net; 7 Department of Radiation Oncology, Montefiore Einstein Comprehensive Cancer Center, Albert Einstein College of Medicine, New York, NY 10461, USA; alshakir@montefiore.prg; 8 Department of Urology, Mater Dei Hospital, 70125 Bari, Italy; michele.battaglia@urologia.uniba.it; 9 Urology Department, Centro Universitario FMABC, Santo Andrè 09060-870, SP, Brazil; willy.baccaglini@einstein.br; 10 Department of Urology, University of Verona, Azienda Ospedaliera Universitaria Integrata di Verona, 37126 Verona, Italy; antoniobenito.porcaro@aovr.veneto.it (A.B.P.); alessandro.antonelli@aovr.veneto.it (A.A.); 11 USC Institute of Urology, Catherine and Joseph Aresty Department of Urology, Keck School of Medicine, University of Southern California, Los Angeles, CA 90007, USA; andre.abreu@med.usc.edu

**Keywords:** RAPN, NIRF, ICG, Renal Cell Carcinoma, minimally invasive surgical procedures

## Abstract

*Background and Objectives*: Partial nephrectomy is the standard treatment for small renal tumors, balancing cancer control with renal function preservation. Robot-assisted partial nephrectomy (RAPN) has improved surgical precision and reduced morbidity. Near-infrared fluorescence (NIRF) imaging with indocyanine green (ICG) improves intraoperative visualization of renal vasculature and tissue perfusion, potentially enabling selective arterial clamping to reduce ischemic injury. This study updates contemporary evidence on NIRF/ICG-guided RAPN, focusing on intraoperative, perioperative, and renal function outcomes. *Materials and Methods*: We systematically queried PubMed, Scopus, and Web of Science databases up to June 2025 for controlled prospective and retrospective studies comparing NIRF/ICG-guided RAPN (selective clamping or zero-ischemia) versus conventional RAPN with main artery clamping in adults with renal masses. Data were synthesized narratively, and random-effects meta-analyses were performed on warm ischemia time (WIT), operative time, estimated blood loss, transfusion rate, length of hospital stay, complication rate, positive surgical margins, and variation in renal function. *Results*: Eleven studies (10 full-text and one abstract), including two randomized controlled trials, encompassing a patient population of 893 patients (403 NIRF/ICG-guided RAPN and 490 conventional RAPN), were included. Ischemia strategies varied between no clamping, selective or super-selective clamping for NIRF/ICG, and main artery clamping for controls. ICG doses ranging from 3 to 7.5 mg or 0.5–7 mL. Most evidence was classified as level 2b or 3b, indicating a moderate to serious risk of bias. Meta-analysis showed that compared to conventional RAPN, NIRF/ICG-guided RAPN was associated with a shorter WIT (MD: −1.30 min, 95% CI: −2.51 to −0.09; *p* = 0.039), with no differences in other outcomes. Renal function favored NIRF/ICG at discharge and short-term follow-up, although the difference was not statistically significant. *Conclusions*: NIRF/ICG reduces WIT during RAPN without increasing perioperative risks. The technique shows promise for better preserving functional outcomes. However, further well-designed, large-scale trials with longer follow-up are needed to confirm these benefits and define clinical indications.

## 1. Introduction

Partial nephrectomy (PN) is the recommended treatment for small renal tumors when technically feasible [1,2] as it ensures cancer control outcomes while maximizing renal function preservation compared to radical nephrectomy [3,4]. The development of minimally invasive approaches, particularly robot-assisted partial nephrectomy (RAPN), has further improved surgical precision, reduced perioperative morbidity, and shortened recovery compared to traditional laparoscopy [5,6]. A key objective in nephron-sparing surgery is minimizing warm ischemia time (WIT), as duration beyond 25 min can lead to irreversible renal damage [7,8]. To mitigate this, techniques such as off-clamp and selective arterial clamping have been introduced. However, these methods can be technically challenging and often lack intraoperative feedback on tissue perfusion [9,10].

Near-infrared fluorescence (NIRF) imaging with indocyanine green (ICG) enhances the visualization of vascular structures, tissue perfusion, and tumor boundaries in real-time. ICG is a water-soluble dye that binds to plasma proteins shortly after intravenous administration and is primarily cleared by the liver. When exposed to near-infrared light, ICG emits fluorescence, allowing surgeons to identify anatomical landmarks and assess tissue perfusion with high accuracy [11,12].

In urologic surgery, NIRF-ICG is a safe, feasible, and useful tool adopted for various purposes, including the identification of ureters, assessment of renal perfusion, detection of sentinel lymph nodes, and evaluation of vascular supply during both oncological and non-oncological procedures [13]. Its ability to enhance visualization without altering the surgical field has made it particularly valuable in minimally invasive and robot-assisted procedures. Specifically, in the context of renal tumors, NIRF-ICG has emerged as a tool to support selective arterial clamping during RAPN, potentially minimizing global renal ischemia and preserving postoperative renal function [13,14].

Despite its growing adoption, the evidence supporting NIRF-guided selective clamping in RAPN remains heterogeneous, with published studies reporting conflicting results regarding its impact and benefit on perioperative and renal function outcomes [15,16,17,18]. A previous systematic review by Veccia et al. and Zhou et al. suggested improved short-term renal function in patients undergoing NIRF/ICG-guided RAPN compared to those who underwent a conventional approach; however, definitive conclusions remain limited due to the small number of included studies [19,20].

The objective of our study was to investigate the role of NIRF/ICG in RAPN by comparing the postoperative, perioperative, and renal function outcomes between NIRF/ICG-guided RAPN with selective clamping or the zero-ischemia technique and conventional RAPN with main artery clamping.

## 2. Materials and Methods

### 2.1. Search Strategy and Selection of Eligible Studies

This systematic review was performed following the Preferred Reporting Items for Systematic Review and Meta-analyses (PRISMA) statement (Supplementary Table S1) [21]. PubMed, Scopus, and Web of Science databases were searched systematically for articles focusing on the use of NIRF with ICG during RAPN in patients with renal masses, up to June 2025. The search string used was as follows: (ICG OR indocyanine green OR near-infrared fluorescence OR NIRF) AND (robot-assisted partial nephrectomy OR RAPN OR robotic partial nephrectomy). The present study was registered with PROSPERO (International Prospective Register of Systematic Reviews) under the registration code CRD420251114676.

Two paired investigators (A.P. and R.O.) independently screened all titles gathered from the literature review to identify potentially eligible studies. They then evaluated abstracts and full-text manuscripts to determine the final set of included ones. Any disagreements were resolved by a third independent author (A.T.), after a collegial discussion to reach a final consensus. Only controlled studies (prospective or retrospective), published in the form of a full-text manuscript or conference proceedings, comparing transperitoneal or retroperitoneal RAPN with selective artery clamping or no clamping using NIRF/ICG and conventional RAPN with main artery clamping in adult patients with renal masses were considered. The PICOT (Population, Intervention, Comparators, Outcomes, Type of study) format [22] summarizes our research and analysis strategy for evaluating the outcome of interest (Table 1). No limitation was applied to the number of patients, outcomes, or duration of follow-up. Systematic or narrative reviews and articles focusing on pediatric patients or other diseases or treatment types, as well as experimental studies, case reports, surveys, editorials, and book chapters, were excluded. Reference lists of relevant and recent systematic reviews were also manually reviewed to identify supplementary studies of interest.

### 2.2. Data Extraction, Quality of Studies, and Risk of Bias Assessment

All data extracted from the included studies were recorded in an electronic database. Collected data included: authors; journal; type and year of publication; country of origin; design of the study; interventions; number of participants; baseline demographics (age [years], sex, body mass index [BMI, Kg/m^2^], Charlson Comorbidity Index [CCI], preoperative estimated glomerular filtration rate [eGFR, mL/min/1.73 m^2^], primary tumor size [cm], R.E.N.A.L. or PADUA score); and outcomes measured (operative time [OT, min], estimated blood loss [EBL, mL], WIT [min], length of hospital stay [LOS, days], rate of overall complications [defined as any Clavien–Dindo grade] and major complications [Clavien–Dindo ≥ III], transfusion rate [defined as any allogeneic red-blood-cell transfusion during the index admission], positive surgical margins [PSM] rate, and eGFR values at discharge, at 1-, 3-, and 6-months follow-up).

All full-text articles were categorized according to their level of evidence using the 2011 system of the Oxford Level of Evidence Working Group [23]. The risk of bias was independently assessed by two paired investigators (A.P. and R.O.) for all full-text included studies using Cochrane tools for comparative randomized and non-randomized studies. The risk of bias assessment was generated with the RoB-2 [23] for randomized controlled trials (RCTs), and the ROBINS-I [24] tool for non-randomized trials. Any disagreements were resolved by a third independent author (A.T.), after a collegial discussion to reach a final consensus.

### 2.3. Statistical Analyses

Random-effects meta-analyses compared NIRF/ICG-guided RAPN with conventional RAPN (no NIRF/ICG). Continuous outcomes (WIT, OT, EBL, LOS, and renal function as absolute eGFR at discharge, 1, 3, and 6 months, plus a short-term 1–3-month composite) were pooled as mean differences (MD) with 95% confidence interval (CIs); dichotomous outcomes (transfusions, overall and major complications, PSM) as odds ratio (OR) with 95% CIs. When medians and interquartile range (IQR) were reported, means and standard deviation (SD) were approximated using established methods (Wan et al.) [25].

For studies reporting both 1- and 3-month eGFR, we present separate timepoint meta-analyses and also a within-study short-term composite under a conservative correlation ρ = 0.50 (with sensitivity at ρ = 0.25 and ρ = 0.75). Between-study variance (τ^2^) was estimated using DerSimonian–Laird (DL), and CIs were computed with Hartung–Knapp (HK). As sensitivity analyses, all models were re-estimated using Restricted Maximum Likelihood (REML) with HK. Heterogeneity was summarized with I^2^ and τ^2^; 95% prediction intervals were inspected where applicable. Leave-one-out influence analyses were run for each endpoint. Small-study effects were explored using funnel plots and Egger’s regression when 10 or more studies were available (acknowledging limited power). For dichotomous outcomes, a continuity correction of 0.5 was applied to single-arm zero cells; double-zero studies were synthesized with continuity-corrected ORs and re-assessed in sensitivity analyses excluding such studies. We prespecified subgroup analyses by ischemia strategy (off-clamp, selective, super-selective), surgical approach (transperitoneal vs. retroperitoneal), and tumor complexity category (low-intermediate vs. high), and repeated random-effects models using REML+HK as a sensitivity analysis to the DL+HK primary specification. Subgroup differences were assessed with the χ^2^ test for interaction (Q_between).

Forest plots display study-specific effects (MD for continuous outcomes; log OR for dichotomous outcomes) with 95% CIs and the random-effects pooled estimate (DL with HK). Square size is proportional to inverse-variance weight; diamonds indicate the pooled effect and its 95% CI. The vertical reference line marks the null (MD = 0; OR = 1). All continuous outcomes are presented as MDs in natural units. Two-sided *p*-values < 0.05 were considered statistically significant. All analyses were conducted in R (version 4.1.2; R Foundation for Statistical Computing, Vienna, Austria) [26].

## 3. Results

### 3.1. Literature Screening

**Figure 1:**
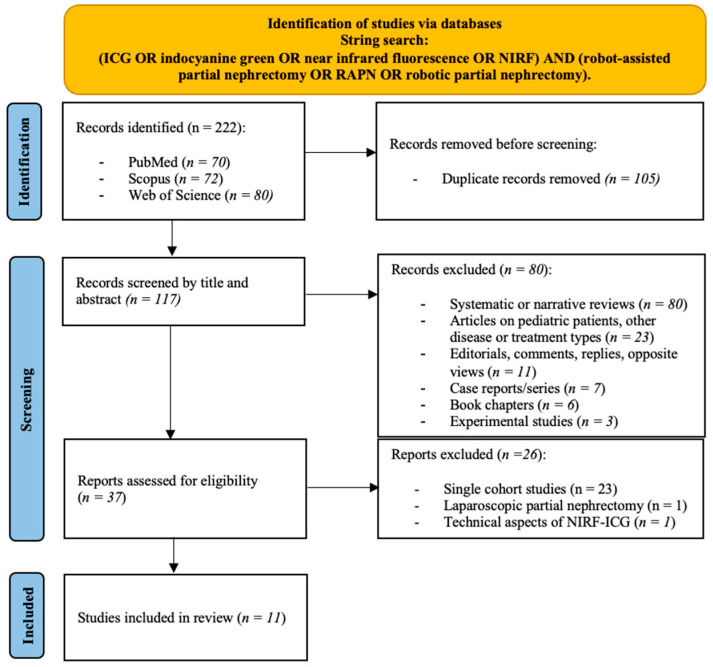
PRISMA (Preferred Reporting Items for Systematic Review and Meta-Analyses) flow diagram for the identification and selection of studies assessing near-infrared fluorescence imaging (NIRF) with indocyanine green (ICG) in robot-assisted partial nephrectomy (RAPN).

The PRISMA diagram presents the results of the literature research (Figure 1). We identified a total of 222 records; after excluding duplicates, 117 records remained for screening based on title and abstract. A total of 37 full-text manuscripts or meeting abstracts were then retrieved and assessed for eligibility, of which 23 single-cohort studies published as abstract or full-text articles, 1 original article only focusing on NIRF/ICG in patients treated with laparoscopic PN, and 1 study only describing technical aspects of NIRF/ICG technique were excluded. Therefore, a total of 11 records (10 full-text manuscripts and 1 conference abstract) published between 2012 and 2025 were included [27,28,29,30,31,32,33,34,35,36,37].

### 3.2. Studies’ Characteristics

The main characteristics of the studies are listed in Table 2. Seven studies were retrospective [28,29,30,31,33,35,37], two were prospective with a matched-pair comparison using retrospectively evaluated controls [27,32], and two were RCTs [34,36]. Sample sizes across studies ranged from 15 to 87 for the NIRF/ICG-guided RAPN group and from 15 to 106 for the conventional RAPN group, with 403 patients receiving NIRF/ICG and 490 not receiving it. Ischemia strategies included no clamping, selective or super-selective clamping for the NIRF/ICG RAPN group, and main artery clamping for the conventional RAPN group. NIRF/ICG was administered intraoperatively at doses ranging from 3 to 7.5 mg or 0.5–7 mL, depending on protocol and institutional practice. Overall, baseline demographics and tumor characteristics were well-balanced between the intervention and control groups. Tumor complexity was reported using R.E.N.A.L. or PADUA scores, with most tumors classified as low to intermediate complexity. Follow-up duration ranged from two weeks to one year, with most studies assessing outcomes at 1–6 months postoperatively.

According to the Oxford 2011 criteria, two studies were classified as level 1b (RCTs) [34,36], two as level 2b (prospective cohorts with matched controls) [27,32], and seven as level 3b (retrospective comparative studies) [28,29,30,31,33,35,37]. The risk of bias assessment was conducted using the ROBINS-I tool for observational studies and the RoB-2 tool for RCTs. Overall, four studies were rated as having a moderate risk of bias [30,33,35,37], four as serious [27,28,31,32], and two RCTs as some concerns [34,36]. The most frequent concerns involved confounding and selective reporting (Table 3, Supplementary Figures S1 and S2).

### 3.3. Main Studies’ Findings

Four studies retrospectively compared patients undergoing NIRF/ICG-guided RAPN with a matched cohort of patients undergoing conventional RAPN, based on patient demographics, preoperative renal function, and tumor complexity. Borofsky et al. [28] investigated outcomes of zero-ischemia RAPN using NIRF/ICG in 34 patients, with 27 (79%) successfully undergoing super-selective clamping. Compared to those who underwent conventional RAPN with main artery clamping (n = 27), these patients had highly comparable outcomes, except for longer OT (256 vs. 212 min, *p* = 0.02), and better short-term renal function preservation (eGFR reduction −1.8% vs. −14.9%, *p* = 0.03). All surgical margins were negative. Bjurlin et al. [29] compared outcomes of patients undergoing NIRF/ICG-guided RAPN with selective arterial clamping (n = 39) to patients undergoing conventional RAPN with non-selective clamping (n = 39). At short-term follow-up, patients in the NIRF/ICG group showed a statistically significantly higher reduction in both absolute (−8.82 vs. −15.6 mL/min, *p* = 0.0473) and percentage (−10.5 vs. −18.4%, *p* = 0.0371) change in eGFR. This contribution was only available in the form of a conference abstract. Harke et al. [30] evaluated the feasibility of NIRF/ICG-guided RAPN in 22 patients, with selective clamping possible in 21 of 22 cases, resulting in no intraoperative complications and a mean WIT of 11.6 min. The authors then compared data of 15 subjects with a matched cohort of 15 patients treated with conventional RAPN with main artery clamping and global ischemia, finding that the selective clamping brought about less eGFR reduction (5.1 vs. 16.1 mL/min, *p* = 0.045). McClintock et al. [31] compared 42 patients undergoing NIRF/ICG-guided RAPN with 42 undergoing RAPN with main artery clamping. The former group showed significantly better renal function at discharge, with smaller reductions in absolute eGFR value (−2.5 vs. −14.0 mL/min; *p* < 0.01) and eGFR percentage change (–1.9 vs. –16.8%; *p* < 0.01). However, differences at 3 months were not statistically significant.

Two studies prospectively collected data and outcomes of patients treated with NIRF/ICG-guided RAPN and then compared them with a matched cohort of retrospectively evaluated controls treated with conventional RAPN. In the study by Krane and colleagues [27] 47 patients who received NIRF/ICG-guided RAPN were matched with 47 patients who received conventional RAPN. Apart from WIT that was significantly shorter in the NIRF/ICG group (15 vs. 17 min, *p* = 0.01), the authors did not find significant differences in terms of PSM (6 vs. 8.5%, *p* = 0.69), complications (4 vs. 15%, *p* = 0.07), and postoperative functional outcomes between cohorts, including percentage change in eGFR at discharge. In the study by Lanchon and colleagues [32], 25 patients who underwent super-selective RAPN guided by NIRF/ICG were matched with 25 patients who underwent early-unclamping RAPN. Super-selective clamping was successful in 77% of cases and demonstrated similar perioperative and oncological outcomes compared to the early unclamping technique. However, it was associated with significantly better eGFR preservation at discharge (0 vs. −15%, *p* = 0.002), 1 (−1 vs. −13%, *p* = 0.01) and 3 months (−2 vs. −12%, *p* = 0.01), improved relative renal function of operated kidney on scintigraphy (46 vs. 40%, *p* = 0.04), and fewer CKD stage ≥3 cases (1 vs. 6, *p* = 0.03).

Three studies had a complete retrospective design without a matching adjustment. Mattevi et al. [33] compared perioperative and functional outcomes of a cohort of 20 patients receiving NIRF/ICG-guided selective clamping RAPN with 42 patients receiving conventional RAPN with main artery clamping. Selective clamping was successful in 75% (n = 15) of NIRF/ICG cases. OT and EBL were similar between groups. No major complications were recorded in the NIRF/ICG-guided RAPN group, while three acute hemorrhages were recorded in the conventional RAPN group. Renal scan analysis showed significantly lower loss of operated kidney eGFR (5.5% vs. 21.5%, *p* = 0.046) and total eGFR (0% vs. 8%, *p* = 0.007) in the NIRF/ICG group. Yang et al. [35] evaluated a series of 127 RAPN cases, including 21 performed with NIRF/ICG use. Compared to patients undergoing conventional RAPN, those undergoing NIRF/ICG-guided RAPN showed longer OT (311 vs. 271 min; *p* = 0.006) but superior eGFR preservation rate at 3-month follow-up (90 vs. 85%; *p* = 0.031). Joffe et al. [37] examined a cohort of 150 patients undergoing both laparoscopic and robotic PN, 58% (n = 87) of whom underwent PN with NIRF/ICG. ICG use was not associated with differences in EBL, WIT, or PSM rates. In multivariable analysis, a tendency towards statistical significance was observed for ICG use and postoperative change in CKD stage (OR: 9.9, *p* = 0.05), with no impact on creatinine change or other perioperative outcomes.

Two RCTs were available. Long et al. [34] conducted the first RCT comparing super selective RAPN using NIRF/ICG to conventional RAPN with early unclamping in patients with a single renal tumor. The authors found no significant difference in operated kidney eGFR reduction, as well as in global eGFR reduction between groups at discharge, 1- and 6-month follow-up, with perioperative outcomes also being comparable. The trial was stopped early due to futility, raising questions about the clinical benefit of this intervention. Finally, Mazzoleni et al. [36] compared ICG fluorescence-guided off-clamp RAPN with preoperative super selective embolization (n = 70) with intraoperative ultrasound on-clamp RAPN without super selective embolization (n = 70). Compared to the latter, the former group showed significantly shorter OT (86.5 vs. 121.8 min, *p* = 0.02), lower EBL (72.8 vs. 214.2 mL, *p* = 0.02), and smaller hemoglobin drop (*p* = 0.04). However, no statistically significant differences were found in serum creatinine at the 1-month follow-up, and complication and transfusion rates were similar.

### 3.4. Meta-Analysis of Perioperative and Postoperative Outcomes

#### 3.4.1. Warm Ischemia Time

**Figure 2:**
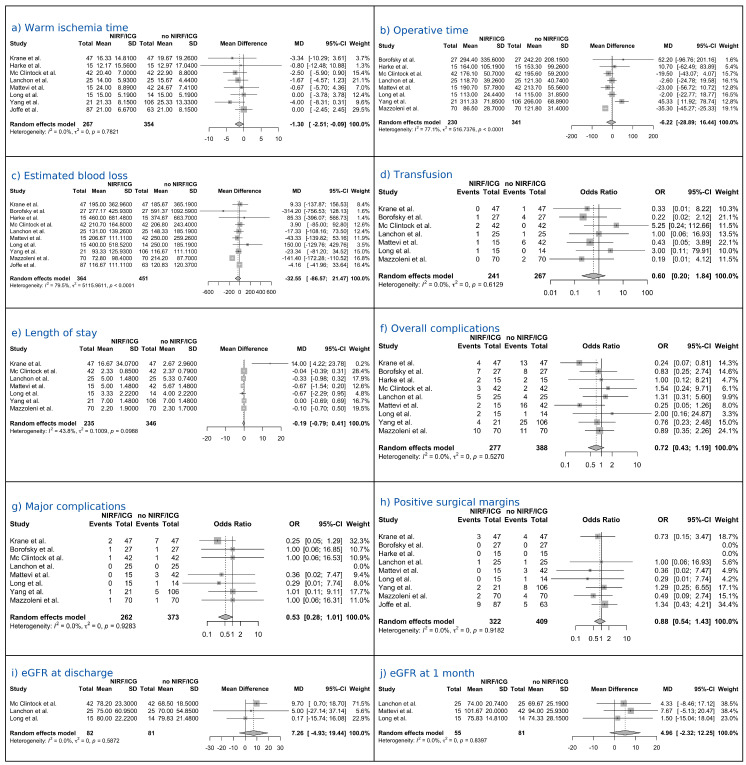

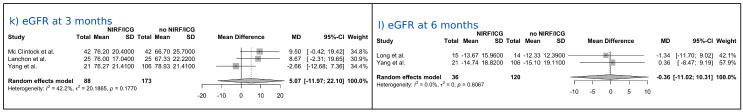
Forest plots of perioperative and postoperative outcomes [27,28,30,31,32,33,34,35,36,37].

Eight studies comprising a total of 621 patients reported complete data on WIT. The random-effects analysis demonstrated that the NIRF/ICG-guided RAPN group had a significantly shorter WIT compared to the control group (no NIRF/ICG RAPN), with a pooled MD of −1.30 min (95% CI: −2.51 to −0.09; *p* = 0.039). Between-study heterogeneity was negligible (I^2^ = 0%; Figure 2a).

#### 3.4.2. Operative Time

Eight studies comprising a total of 571 patients reported complete data on OT. The random-effects analysis showed a pooled MD of −6.22 min (95% CI: −28.94 to 16.44; *p* = 0.537), indicating no statistically significant difference between the NIRF/ICG-guided RAPN and the control group. Considerable heterogeneity was observed among the studies (I^2^ = 77.1%; Figure 2b).

#### 3.4.3. Estimated Blood Loss

Ten studies comprising a total of 815 patients reported complete data on EBL. The random-effects analysis showed a pooled MD of −32.55 mL (95% CI: −86.57 to 21.47; *p* = 0.206), indicating no statistically significant difference between the NIRF/ICG-guided RAPN and the control group. Substantial heterogeneity was observed among the studies (I^2^ = 79.5%; Figure 2c).

#### 3.4.4. Transfusions

Seven studies comprising a total of 508 patients reported complete data on the transfusion rate. The random-effects analysis estimated an OR of 0.60 (95% CI: 0.20 to 1.84; *p* = 0.306), indicating no statistically significant difference between the NIRF/ICG-guided RAPN group and the control group. No heterogeneity was observed among the studies (I^2^ = 0%; Figure 2d).

#### 3.4.5. Length of Hospital Stay

Seven studies comprising a total of 581 patients reported complete data on LOS. The random-effects analysis showed a pooled MD of −0.19 days (95% CI: −0.79 to 0.41; *p* = 0.478), indicating no statistically significant difference in hospital stay duration between the NIRF/ICG-guided RAPN group and the control group. Moderate heterogeneity was observed among studies (I^2^ = 43.8%; Figure 2e).

#### 3.4.6. Complications

Nine studies comprising a total of 665 patients reported complete data on overall complication rates. The random-effects analysis estimated an OR of 0.72 (95% CI: 0.43 to 1.19; *p* = 0.168), indicating no statistically significant difference between the NIRF/ICG-guided RAPN group and the control group. No heterogeneity was detected among studies (I^2^ = 0%; Figure 2f). Eight studies comprising a total of 635 patients reported complete data on major complications (Clavien–Dindo ≥ III) rates. The random-effects analysis estimated an OR of 0.53 (95% CI: 0.28 to 1.01; *p* = 0.053), indicating lower odds of major complications with NIRF/ICG-guided RAPN compared with controls with a tendency towards statistical significance. No heterogeneity was detected among studies (I^2^ = 0%; Figure 2g).

#### 3.4.7. Positive Surgical Margins

Nine studies comprising a total of 731 patients reported complete data on PSM. The random-effects analysis estimated an OR of 0.88 (95% CI: 0.54 to 1.43; *p* = 0.552), indicating no statistically significant difference between the NIRF/ICG-guided RAPN group and the control group. No heterogeneity was observed among the studies (I^2^ = 0; Figure 2h).

#### 3.4.8. Renal Function

Three studies comprising a total of 163 patients reported complete data on eGFR at discharge. The random-effects analysis yielded an MD of 7.26 mL/min/1.73 m^2^ (95% CI: −4.93 to 19.44; *p* = 0.125), indicating no statistically significant difference between the NIRF/ICG-guided RAPN group and the control group. No heterogeneity was observed between the studies (I^2^ = 0%; Figure 2i). Three studies comprising a total of 136 patients reported complete data on eGFR at 1-month follow-up. The random-effects analysis yielded a pooled MD of 4.96 mL/min/1.73 m^2^ (95% CI: −2.32 to 12.25; *p* = 0.099), indicating no statistically significant difference between the NIRF/ICG-guided RAPN group and the control group. No heterogeneity was observed between the studies (I^2^ = 0%; Figure 2j). Three studies, comprising a total of 261 patients, reported complete data on eGFR at the 3-month follow-up. The random-effects analysis yielded a pooled MD of 5.07 mL/min/1.73 m^2^ (95% CI: −11.97 to 22.10; *p* = 0.329), indicating no statistically significant difference between the NIRF/ICG-guided RAPN group and the control group. Moderate heterogeneity was observed among studies (I^2^ = 42.2%; Figure 2k). Two studies, comprising a total of 156 patients, reported complete data on eGFR at the 6-month follow-up. The random-effects analysis yielded an MD of −0.36 mL/min/1.73 m^2^ (95% CI: −11.02 to 10.31; *p* = 0.745), indicating no statistically significant difference between the NIRF/ICG-guided RAPN group and the control group. No heterogeneity was observed among the studies (I^2^ = 0%; Figure 2l).

#### 3.4.9. Sensitivity and Subgroup Analyses

Sensitivity analyses using REML+HK yielded pooled estimates and inferences that were concordant with those from the primary DL+HK models (Supplementary Table S2). For dichotomous endpoints, handling of zero cells (single-arm continuity correction and double-zero continuity-corrected ORs), as well as exclusion of double-zero studies, did not materially alter conclusions (Supplementary Table S3). When pooling short-term renal function (1–3 months) with an assumed correlation of 0.50, the results were similar to those of the single-time analyses. Sensitivity analyses using correlations of 0.25 and 0.75 did not materially change the conclusions (Supplementary Table S4). Leave-one-out diagnostics did not identify influential single studies (Supplementary Table S5), and where evaluable (k ≥ 10), Egger’s tests did not suggest small-study bias (Supplementary Table S6).

In pre-specified subgroup analyses, we found no consistent evidence that the effect of NIRF/ICG guidance varied by ischemia-sparing strategy, surgical approach, or tumor complexity. Tests for interaction (Q_between from random-effects subgroup models with HK) were non-significant across most outcomes, and nominal signals were not reproducible across endpoints or sensitivity models (REML). Overall, these findings corroborate the primary analyses—namely, a modest reduction in WIT with NIRF/ICG guidance, without statistically significant differences in other perioperative or renal function outcomes (Supplementary Tables S7–S9).

## 4. Discussion

NIRF imaging with ICG is an intraoperative technique that enhances visualization of renal vasculature and tissue perfusion [13]. Its real-time feedback potentially aids surgeons in performing more precise selective arterial clamping during RAPN [14,18]. Preoperative assessment of renal anatomy traditionally relies on conventional imaging such as computed tomography and magnetic resonance imaging, which provide detailed anatomical information but lack functional and perfusion data [38]. Intraoperative ultrasound offers real-time imaging to delineate tumor margins, but its use can be limited by operator dependency and tissue penetration [39]. More recently, 3D reconstructed models have gained popularity, allowing for enhanced spatial understanding and surgical planning, yet these require specialized software and may prolong preoperative preparation [40,41]. Within this context, NIRF/ICG integrates functional perfusion mapping during surgery, complementing these modalities by providing dynamic vascular and tissue viability information that can refine ischemia management.

In this systematic review and meta-analysis, we investigated the role of NIRF imaging with ICG in RAPN, focusing on perioperative, postoperative, and renal function outcomes. Our findings contribute to the growing body of evidence on the clinical utility of NIRF/ICG-guided selective or zero ischemia techniques compared to standard main artery clamping. We synthesized data from 11 studies, including RCTs and observational cohorts, which independently revealed heterogeneous perioperative and postoperative outcomes, questioning the real benefits of NIRF/ICG-guided RAPN. When available data from included studies were pooled, the meta-analysis showed a modest but statistically significant reduction in WIT in favor of NIRF/ICG guidance. Minimizing WIT is crucial, as prolonged ischemia is a known risk factor for irreversible renal damage [8]. Other perioperative and postoperative outcomes, such as OT, EBL, transfusions, and LOS, were comparable between groups. This suggests that the integration of NIRF/ICG imaging does not negatively impact surgical efficiency or safety, even if it requires additional intraoperative steps.

Regarding postoperative morbidity and oncologic safety, complication rates and PSM incidence were similar between NIRF/ICG-guided and conventional RAPN. Interestingly, when only the major complications rate was evaluated, a lower odds ratio was demonstrated in the NIRF/ICG-guided group compared with controls, with a trend towards statistical significance. This is reassuring and confirms that enhanced visualization techniques do not compromise oncologic control or may also reduce the risk of adverse events.

Finally, renal functional outcomes, measured as eGFR values at discharge, 1, 3, and 6 months of follow-up, showed a trend favoring NIRF/ICG-guided RAPN in the short term. However, differences did not achieve statistical significance. Previous analyses performed by Veccia et al. [19] and Zhou et al. [20] showed statistically significantly higher pooled eGFR values at short-term (1–3 months) follow-up in NIRF/ICG patients. In our contribution, the inclusion of the study by Yang et al. [35], which found no absolute differences in 3-month eGFR values between the two groups, likely contributed to the lack of statistical significance. However, our pooled results are consistent with the signal coming from randomized evidence. Notably, one RCT (EMERALD) was stopped early for futility, finding no clinically meaningful functional advantage despite protocolized selective/zero-ischemia guidance [34], and the other RCT did not demonstrate superior postoperative renal function at early follow-up [36]. Together, these trials suggest that shorter ischemia times do not automatically translate into measurable global eGFR benefits at the studied timepoints, particularly in cohorts with relatively preserved baseline renal function and limited long-term follow-up.

The routine application of NIRF/ICG in all RAPN cases remains to be validated, given the current limitations in evidence. Its use should be individualized based on the surgeon’s expertise, tumor anatomy, and institutional resources. Our subgroup exploration did not identify reproducible effect modification by ischemia strategy, surgical approach, or tumor complexity. Interaction tests were non-significant across most endpoints. Still, biologically plausible niches—such as totally endophytic or hilar masses, solitary kidney, multiple renal tumors, pre-existing chronic renal insufficiency—may benefit from better segmental perfusion mapping and targeted clamping. In these cases, precise identification of perfused parenchyma can inform selective clamping strategies to minimize ischemic damage. Most of the included studies lack specific information regarding the surgical approach of RAPN (transperitoneal vs. retroperitoneal). Given that the retroperitoneal approach is increasingly favored in specific clinical scenarios due to its direct access to the kidney and potentially reduced morbidity [42], future research may investigate the role of NIRF/ICG within this context. Notably, a recent study explored the use of novel fluorescent tracers to assist retroperitoneal RAPN, suggesting promising avenues for improved vascular mapping and tissue perfusion assessment in this approach, with favorable outcomes, successful renal function preservation, and precise tumor excision [43]. We consider these hypothesis-generating signals rather than definitive indications.

Our study has limitations. The differences among included studies in terms of ischemia management (selective, super-selective, or zero ischemia), institutional protocols and ICG dosing, and surgeons’ experience may influence perioperative outcomes, such as OT and EBL. Additionally, differences in tumor complexity, patient comorbidities, and baseline renal function could confound results despite matched cohort designs. The limited sample sizes in some studies and relatively short follow-up periods limit conclusions about the sustained benefits to renal function and oncological safety. All these limitations should be considered when reading the results of the analyses performed, which should be interpreted with caution. Finally, the overall quality of evidence was moderate, with most studies being retrospective in design. Priorities for future research include standardized selective-clamping protocols, multicenter RCTs with rigorous design, adequately powered, with concealed allocation, standardized ICG dosing/administration, longer follow-up to evaluate durability of renal function, late complications, and oncologic safety, and patient-relevant renal endpoints (e.g., eGFR of the operated kidney, CKD stage migration, ≥25% eGFR decline), alongside prespecified analyses of anatomy-driven complexity. These steps will clarify whether NIRF/ICG confers clinically meaningful renal protection in specific patient subsets and inform the cost-effective and standardized implementation.

## 5. Conclusions

In contemporary RAPN, NIRF/ICG guidance is associated with a small, consistent reduction in WIT without increasing perioperative morbidity or compromising oncologic outcomes. Although current evidence suggests a positive trend towards improved early renal function preservation, meta-analyses did not find statistically significant differences in eGFR values at discharge and during follow-up; thus, definitive benefits remain to be established, including those in anatomically or physiologically high-risk scenarios. Future protocol-standardized, adequately powered RCTs with extended follow-up and patient-centered renal endpoints are essential to optimize the role of NIRF/ICG in nephron-sparing surgery, thus defining who may benefit and how this technology should be used in routine practice.

## Figures and Tables

**Table 1:** PICOT (Population, Intervention, Comparators, Outcomes, Type of studies) format.

POPULATION (P)	Adults (age ≥ 18 years) with a renal mass undergoing partial nephrectomy.
INTERVENTION (I)	Transperitoneal or retroperitoneal robot-assisted partial nephrectomy (RAPN) with selective artery clamping or no clamping guided by near-infrared imaging (NIRF) with indocyanine green (ICG).
COMPARATOR (C)	Transperitoneal or retroperitoneal RAPN with main artery clamping.
OUTCOME (O)	Any surgical outcomes, including perioperative (e.g., operative time, blood loss, ischemia time), and postoperative (complications, renal function preservation, length of in-hospital stay, oncological outcomes).
TYPE OF STUDY (T)	Comparative studies (prospective and retrospective) published as a full-text manuscript or conference abstract, with no restriction on sample size or follow-up duration.

**Table 2:** Main characteristics of the included studies.

Study	Design	Cases/Controls	Clamp Method/ Ischemia Type	ICG Dose	Sample Size	Sex (Males)	BMI	CCI	Tumor Size (cm)	Laterality (Right)	**Complexity Score**	**Follow-Up**
Krane et al. Urology USA, 2012 [27]	Prospective enrolled cases matched with retrospective evaluated controls	NIRF-RAPN	Main artery clamping (global ischemia) or selective clamping or no clamping (zero-ischemia)	5–7.5 mg	47	29 (62%)	28.9	NA	2.7	24 (51%)	RENAL 4–6: 31 (66%) RENAL 7–9: 15 (32%) RENAL ≥10: 1 (2%) PADUA 6–7: 30 (64%) PADUA 8–9: 10 (21%) PADUA ≥10: 7 (15%)	5 months
		no NIRF-RAPN	Main artery clamping (global ischemia) or no clamping (zero-ischemia)	__	47	24 (51%)	30.2	NA	2.6	25 (53%)	RENAL 4–6: 29 (62%) RENAL 7–9: 16 (34%) RENAL ≥10 0 (0%) PADUA 6–7: 22 (49%) PADUA 8–9: 19 (37%) PADUA ≥10: 4 (10%)	5 months
Borofsky et al. BJU Int USA, 2013 [28]	Retrospective matched-paired study	NIRF-RAPN	No clamping (zero ischemia)	7.5 mg	27	16 (59%)	28.9	NA	2.79	14 (52%)	RENAL: 8 (5–10)	13.5 days
		no NIRF-RAPN	Main artery clamping (global ischemia)	__	27	15 (56%)	30.1	NA	3.24	16 (59%)	RENAL: 8 (5–10)	12.7 days
Bjurlin et al. J Urol USA, 2013 [29]	Retrospective matched-paired study	NIRF-RAPN	Selective clamping	NA	39	28	29.3	NA	2.8	NA	NA	NA
		no NIRF-RAPN	Non-selective clamping	__	39	NA	NA	NA	2.7	NA	NA	NA
Harke et al. World J Urol Germany, 2013 [30]	Retrospective matched-paired study	NIRF-RAPN	Selective clamping	5 mg	15	NA	28.9	1.3	3.71	NA	RENAL 4–6: 2 (13%) RENAL 7–9: 10 (67%) RENAL ≥10: 3 (20%)	NA
		no NIRF-RAPN	Main artery clamping (global ischemia)	__	15	NA	27.7	1.5	3.24	NA	RENAL 4–6: 2 (13%) RENAL 7–9: 10 (67%) RENAL ≥10: 3 (20%)	NA
McClintock et al. Urology USA, 2014 [31]	Retrospective matched-paired study	NIRF-RAPN	Selective clamping	5–7.5 mg	42	30	29	NA	2.81	NA	RENAL: 6.67 (1.75)	3 months
		no NIRF-RAPN	Main artery clamping (global ischemia)	__	42	26	28.2	NA	2.97	NA	RENAL: 7.35 (1.94)	3 months
Lanchon et al. Int Braz J Urol France, 2018 [32]	Prospective enrolled cases matched with retrospective evaluated controls	NIRF-RAPN	Super selective clamping (zero ischemia)	0.5–2 cc	25	22 (88%)	27	5	3.0	17 (68%)	RENAL 4–6: 7 (28%) RENAL 7–9: 11 (44%) RENAL ≥10: 7 (28%) PADUA 6–7: 7 (28%) PADUA 8–9: 11 (44%) PADUA ≥10: 7 (28%)	3 months
		no NIRF-RAPN	Early-unclamping	__	25	17 (68%)	25	5	4.0	14 (56%)	RENAL 4–6: 5 (20%) RENAL 7–9: 13 (52%) RENAL ≥10: 7 (28%) PADUA 6–7: 4 (16%) PADUA 8–9: 13 (52%) PADUA ≥10: 8 (32%)	3 months
Mattevi et al. J Robot Surg Italy, 2019 [33]	Retrospective comparative study	NIRF-RAPN	Selective artery clamping	5 mg	15	12 (75%)	27	2	4.0	10 (67%)	PADUA 6–7: 6 (40%) PADUA 8–9: 5 (33.3%) PADUA ≥10: 4 (26.7%)	1 month
		no NIRF-RAPN	Main artery clamping (global ischemia)	__	42	27 (64%)	27	3	4.0	21 (50%)	PADUA 6–7: 18 (43%) PADUA 8–9: 17 (40%) PADUA ≥10: 7 (17%)	1 month
Long et al. Eur Uro Focus France, 2022 [34]	Randomized clinical trial	NIRF-RAPN	Super selective clamping (zero ischemia)	5 cc	15	10 (67%)	26.7	2	2.6	9 (60%)	RENAL: 7 (6–9) PADUA: 8 (7–10)	6 months
		no NIRF-RAPN	Early unclamping	__	14	9 (64%)	26.5	2	3.0	7 (50%)	RENAL 8 (6–9) PADUA 7.5 (7–9)	6 months
Yang et al. Cancers Taiwan, 2022 [35]	Retrospective comparative study	NIRF-RAPN	Main artery clamping	3–5 mL	21	12 (57%)	26.5	NA	3.3	13 (62%)	RENAL: 8 (6–8)	6 months
		no NIRF-RAPN	Main artery clamping	__	106	60 (57%)	25.1	NA	2.9	63 (59%)	RENAL: 8 (6–9)	6 months
Mazzoleni et al. BJU Compass Italy, 2023 [36]	Randomized clinical trial	NIRF-RAPN	Super selective preoperative embolization—no clamping	NA	70	NA	25	NA	2.4	33 (47%)	RENAL: 8.3 (3.4)	1 month
		no NIRF-RAPN	No preoperative embolization— main artery clamping and intraoperative ultrasonography	__	70	NA	26	NA	2.5	34 (49%)	RENAL: 7.9 (4.1)	1 month
Joffe et al. J Robot Surg USA, 2025 [37]	Retrospective comparative study	NIRF-PN	Main artery clamping (hilar) or selective clamping	7 mL	87	58 (67%)	NA	NA	3.1	NA	NA	6–12 months
		no NIRF-PN	Main artery clamping (hilar)	__	63	40 (63%)	NA	NA	2.7	NA	NA	6–12 months

**Table 3:** Summary of the included studies published as full-text manuscripts: design, level of evidence, and risk of bias assessment.

Author (Year)	Design	LE (Oxford 2011)	Risk of Bias (Tool)
Krane et al. [27]	Observational, matched cohort	2b	Serious (ROBINS-I)
Borofsky et al. [28]	Observational, matched cohort	3b	Serious (ROBINS-I)
Harke et al. [30]	Observational, matched cohort	3b	Moderate (ROBINS-I)
McClintock et al. [31]	Observational, matched cohort	2b	Serious (ROBINS-I)
Lanchon et al. [32]	Observational, matched cohort	2b	Serious (ROBINS-I)
Mattevi et al. [33]	Observational, comparative	3b	Moderate (ROBINS-I)
Long et al. [34]	RCT	1b	Some concerns (RoB-2)
Yang et al. [35]	Observational, comparative	3b	Moderate (ROBINS-I)
Mazzoleni et al. [36]	RCT	1b	Some concerns (RoB-2)
Joffe et al. [37]	Observational, comparative	3b	Moderate (ROBINS-I)

## Data Availability

All original data were available in the original article included in the review. The full dataset and code for statistical analyses are available upon request from the corresponding author.

## Supplementary Materials

The following supporting information can be downloaded at: https://www.mdpi.com/article/10.3390/medicina61101735/s1, Supplementary Table S1: PRISMA 2020 checklist; Supplementary Table S2: Sensitivity analyses (REML + Hartung–Knapp); Supplementary Table S3: Dichotomous outcomes—sensitivity excluding double-zero studies (DL + HK); Supplementary Table S4: Short-term renal function composite (1–3 months) with correlation assumptions; Supplementary Table S5: Leave-one-out influence—pooled estimate range; Supplementary Table S6: Small-study effects (Egger’s regression) where k ≥ 10; Supplementary Table S7: Subgroup analyses by ischemia-sparing strategy (DL+HK and REML+HK); Supplementary Table S8: Subgroup analyses by surgical approach (DL+HK and REML+HK); Supplementary Table S9: Subgroup analyses by tumor complexity (DL+HK and REML+HK); Supplementary Figure S1: Risk of bias (ROBINS-I) for non RCTs; Supplementary Figure S2: Risk of bias (RoB 2) for RCTs.
