# Supplementary material for: The Role of Near-Infrared Fluorescence with Indocyanine Green in Robot-Assisted Partial Nephrectomy: Results from an Updated Systematic Review and Meta-Analyses of Controlled Studies
Source: Medicina (Kaunas). 2025 Sep 24;61(10):1735. doi: 10.3390/medicina61101735 (PMC12566515; doi:10.3390/medicina61101735)

Supplementary Table S1. PRISMA 2020 checklist.

Section and Topic	Item #	Checklist item	Location where item is reported
**TITLE**			
Title	1	Identify the report as a systematic review.	Page 1, lines 2-4
**ABSTRACT**			
Abstract	2	See the PRISMA 2020 for Abstracts checklist.	Pages 1-2, lines 32-60
**INTRODUCTION**			
Rationale	3	Describe the rationale for the review in the context of existing knowledge.	Page 2, lines 65-95
Objectives	4	Provide an explicit statement of the objective(s) or question(s) the review addresses.	Page 3, lines 96-99
**METHODS**			
Eligibility criteria	5	Specify the inclusion and exclusion criteria for the review and how studies were grouped for the syntheses.	Page 3, lines 115-126 and Table 1
Information sources	6	Specify all databases, registers, websites, organisations, reference lists and other sources searched or consulted to identify studies. Specify the date when each source was last searched or consulted.	Page 3, lines 104-106
Search strategy	7	Present the full search strategies for all databases, registers and websites, including any filters and limits used.	Page 3, lines 106-108
Selection process	8	Specify the methods used to decide whether a study met the inclusion criteria of the review, including how many reviewers screened each record and each report retrieved, whether they worked independently, and if applicable, details of automation tools used in the process.	Page 3, lines 110-114
Data collection process	9	Specify the methods used to collect data from reports, including how many reviewers collected data from each report, whether they worked independently, any processes for obtaining or confirming data from study investigators, and if applicable, details of automation tools used in the process.	Page 3, lines 111-115
Data items	10a	List and define all outcomes for which data were sought. Specify whether all results that were compatible with each outcome domain in each study were sought (e.g. for all measures, time points, analyses), and if not, the methods used to decide which results to collect.	Pages 3-4, lines 131-141
	10b	List and define all other variables for which data were sought (e.g. participant and intervention characteristics, funding sources). Describe any assumptions made about any missing or unclear information.	NA
Study risk of bias assessment	11	Specify the methods used to assess risk of bias in the included studies, including details of the tool(s) used, how many reviewers assessed each study and whether they worked independently, and if applicable, details of automation tools used in the process.	Page 4, lines 143-149
Effect measures	12	Specify for each outcome the effect measure(s) (e.g. risk ratio, mean difference) used in the synthesis or presentation of results.	Page 4, lines 152-156

Section and Topic	Item #	Checklist item	Location where item is reported
Synthesis methods	13a	Describe the processes used to decide which studies were eligible for each synthesis (e.g. tabulating the study intervention characteristics and comparing against the planned groups for each synthesis (item #5)).	NA
	13b	Describe any methods required to prepare the data for presentation or synthesis, such as handling of missing summary statistics, or data conversions.	Page 4, lines 156-157
	13c	Describe any methods used to tabulate or visually display results of individual studies and syntheses.	Page 4, lines 175-179
	13d	Describe any methods used to synthesize results and provide a rationale for the choice(s). If meta-analysis was performed, describe the model(s), method(s) to identify the presence and extent of statistical heterogeneity, and software package(s) used.	Page 4, lines 145-164
	13e	Describe any methods used to explore possible causes of heterogeneity among study results (e.g. subgroup analysis, meta-regression).	Page 4, lines 163-164, and 169-174
	13f	Describe any sensitivity analyses conducted to assess robustness of the synthesized results.	Page 4, lines 162-163
Reporting bias assessment	14	Describe any methods used to assess risk of bias due to missing results in a synthesis (arising from reporting biases).	NA
Certainty assessment	15	Describe any methods used to assess certainty (or confidence) in the body of evidence for an outcome.	NA
**RESULTS**			
Study selection	16a	Describe the results of the search and selection process, from the number of records identified in the search to the number of studies included in the review, ideally using a flow diagram.	Page 5, lines 186-194 and Figure 1
	16b	Cite studies that might appear to meet the inclusion criteria, but which were excluded, and explain why they were excluded.	Page 5, lines 186-194 and Figure 1
Study characteristics	17	Cite each included study and present its characteristics.	Pages 5-6-7-8, lines 200-323 and Table 2
Risk of bias in studies	18	Present assessments of risk of bias for each included study.	Page 6, lines 216-221, Table 3 and Supplementary materials
Results of individual studies	19	For all outcomes, present, for each study: (a) summary statistics for each group (where appropriate) and (b) an effect estimate and its precision (e.g. confidence/credible interval), ideally using structured tables or plots.	Pages 8-9, lines 325-390
Results of syntheses	20a	For each synthesis, briefly summarise the characteristics and risk of bias among contributing studies.	Supplementary materials (fig 1 and 2)
	20b	Present results of all statistical syntheses conducted. If meta-analysis was done, present for each the summary estimate and its	Pages 8-9,

Section and Topic	Item #	Checklist item	Location where item is reported
		precision (e.g. confidence/credible interval) and measures of statistical heterogeneity. If comparing groups, describe the direction of the effect.	lines 325-390, Figure 2
	20c	Present results of all investigations of possible causes of heterogeneity among study results.	Pages 8-9, lines 325-390
	20d	Present results of all sensitivity analyses conducted to assess the robustness of the synthesized results.	Page 11, lines 395-405
Reporting biases	21	Present assessments of risk of bias due to missing results (arising from reporting biases) for each synthesis assessed.	Supplementary materials
Certainty of evidence	22	Present assessments of certainty (or confidence) in the body of evidence for each outcome assessed.	NA
**DISCUSSION**			
Discussion	23a	Provide a general interpretation of the results in the context of other evidence.	Pages 11-12, lines 428-462
	23b	Discuss any limitations of the evidence included in the review.	Pages 12-13, lines 481-490
	23c	Discuss any limitations of the review processes used.	Pages 12-13, lines 481-490
	23d	Discuss implications of the results for practice, policy, and future research.	Page 12-13, lines 463-480 and 490-498
**OTHER INFORMATION**			
Registration and protocol	24a	Provide registration information for the review, including register name and registration number, or state that the review was not registered.	Page 3, lines 108-110
	24b	Indicate where the review protocol can be accessed, or state that a protocol was not prepared.	Page 3, lines 108-110
	24c	Describe and explain any amendments to information provided at registration or in the protocol.	NA
Support	25	Describe sources of financial or non-financial support for the review, and the role of the funders or sponsors in the review.	Page 13-14, lines 527-533
Competing interests	26	Declare any competing interests of review authors.	Page 13-14, lines 527-533
Availability of data, code and other materials	27	Report which of the following are publicly available and where they can be found: template data collection forms; data extracted from included studies; data used for all analyses; analytic code; any other materials used in the review.	Page 13-14, lines 527-533

Supplementary Table S2. Sensitivity analyses (REML + Hartung–Knapp).

Outcome	k	Effect	Pooled	CI_low	CI_high	p_value	I2	tau2	PI_low	PI_high
WIT (min)	8	MD	-1.30	-2.51	-0.09	0.039	0.0%	0.00	-2.96	0.37
OT (min)	8	MD	-6.94	-29.36	15.48	0.488	77.1%	397.29	-60.71	46.83
EBL (mL)	10	MD	-37.23	-87.67	13.21	0.129	79.4%	2456.28	-163.35	88.90
LOS (days)	7	MD	-0.14	-0.54	0.26	0.430	43.8%	0.00	-0.46	0.18
eGFR at discharge	3	MD	7.26	-4.93	19.44	0.125	0.0%	0.00	-42.07	56.58
eGFR at 1 month	3	MD	4.96	-2.32	12.25	0.099	0.0%	0.00	-46.50	56.43
eGFR at 3 months	3	MD	3.62	-14.80	22.04	0.487	31.1%	10.02	-62.92	70.16
eGFR at 6 months	2	MD	-0.13	-2.25	1.98	0.567	0.0%	0.00	-50.69	50.42
Transfusions	7	OR	0.59	0.22	1.56	0.233	0.0%	0.00	0.15	2.36
Overall complications	9	OR	0.72	0.43	1.19	0.168	0.0%	0.00	0.41	1.24
Major complications (Clavien ≥III)	8	OR	0.58	0.33	1.00	0.050	0.0%	0.00	0.19	1.78
PSM	9	OR	0.91	0.65	1.29	0.564	0.0%	0.00	0.41	2.01

Supplementary Table S3. Dichotomous outcomes — sensitivity excluding double-zero studies (DL + HK).

Outcome	k	Effect	Pooled	CI_low	CI_high	p_value	I2	tau2	PI_low	PI_high
Transfusion	7	OR	0.59	0.22	1.56	0.233	0.0%	0.00	0.15	2.36
Overall complications	9	OR	0.72	0.43	1.19	0.168	0.0%	0.00	0.41	1.24
Major complications (Clavien ≥III)	7	OR	0.56	0.30	1.04	0.062	0.0%	0.00	0.16	1.89
PSM	7	OR	0.91	0.59	1.40	0.610	0.0%	0.00	0.38	2.20

Supplementary Table S4. Short-term renal function composite (1–3 months) with correlation assumptions.

Model

Assumption	k	DL_mu	DL_CI_low	DL_CI_high	DL_p	REML_mu	REML_CI_low	REML_CI_high	I2_DL	I2_REML
rho = 0.50	1	3.99	nan	nan	nan	3.99	nan	nan	0.00	0.0%
rho = 0.25	1	4.36	nan	nan	nan	4.36	nan	nan	0.00	0.0%
rho = 0.75	1	3.48	nan	nan	nan	3.48	nan	nan	0.00	0.0%

Supplementary Table S5. Leave-one-out influence — pooled estimate range.

Outcome	k	Effect	LOO_min	LOO_max
WIT (min)	8	MD	-1.84	-1.01
OT (min)	8	MD	-15.60	-0.44
EBL (mL)	10	MD	-39.29	-10.62
LOS (days)	7	MD	-0.25	-0.12
eGFR at discharge	3	MD	1.12	9.36
eGFR at 1 month	3	MD	3.27	6.00
eGFR at 3 months	3	MD	-2.16	9.46
Transfusion	7	OR	0.46	0.78
Overall complications	9	OR	0.67	0.86
Major complications (Clavien ≥III)	8	OR	0.51	0.83
PSM	9	OR	0.76	1.02

Supplementary Table S6. Small-study effects (Egger's regression) where k ≥ 10.

Outcome	k	Intercept	SE	t	p_value
EBL (mL)	10	0.279	0.778	0.359	0.729

Supplementary Table S7. Subgroup analyses by ischemia-sparing strategy (DL+HK and REML+HK).

Outcome	Subgroup	k	Effect (DL+HK)	p (DL+HK)	Effect (REML+HK)	p (REML+HK)	I² (DL)	Test for interaction
WIT (min)	selective	4	-2.06 [-6.20; 2.07]	0.211	-2.06 [-6.20; 2.07]	0.211	0.0%	Q=0.48, df=2, p=0.788
WIT (min)	super-selective	2	-1.05 [-15.98; 13.88]	0.536	-1.05 [-15.98; 13.88]	0.536	0.0%	Q=0.48, df=2, p=0.788
WIT (min)	mixed	2	-1.59 [-26.47; 23.28]	0.566	-0.98 [-14.80; 12.84]	0.534	60.0%	Q=0.48, df=2, p=0.788
OT (min)	selective	3	-18.61 [-59.62; 22.40]	0.190	-18.61 [-59.62; 22.40]	0.190	0.0%	Q=29.89, df=4, p=0.000
OT (min)	super-selective	2	-2.28 [-100.56; 96.00]	0.817	-2.28 [-100.56; 96.00]	0.817	0.0%	Q=29.89, df=4, p=0.000
EBL (mL)	selective	4	-11.82 [-108.10; 84.46]	0.722	-11.82 [-108.10; 84.46]	0.722	0.0%	Q=41.51, df=4, p=0.000
EBL (mL)	super-selective	2	9.79 [-773.70; 793.27]	0.900	-1.85 [-562.79; 559.08]	0.973	17.1%	Q=41.51, df=4, p=0.000
EBL (mL)	mixed	2	-9.90 [-215.04; 195.25]	0.650	-9.90 [-215.04; 195.25]	0.650	0.0%	Q=41.51, df=4, p=0.000

LOS (days)	selective	3	-0.06 [-7.21; 7.10]	0.975	-0.11 [-0.83; 0.60]	0.569	79.5%	Q=0.80, df=3, p=0.850
LOS (days)	super-selective	2	-0.38 [-4.28; 3.53]	0.435	-0.38 [-4.28; 3.53]	0.435	0.0%	Q=0.80, df=3, p=0.850
eGFR at discharge (mL/min/1.73 m²)	super-selective	2	1.12 [-91.31; 93.55]	0.903	1.12 [-91.31; 93.55]	0.903	0.0%	Q=0.99, df=1, p=0.319
eGFR at 1 month (mL/min/1.73 m²)	super-selective	2	3.27 [-62.32; 68.87]	0.640	3.27 [-62.32; 68.87]	0.640	0.0%	Q=0.28, df=1, p=0.597
Short-term eGFR (1–3 months) (mL/min/1.73 m²)	selective	2	8.81 [-42.03; 59.66]	0.271	8.81 [-42.03; 59.66]	0.271	0.0%	Q=3.13, df=2, p=0.210
Short-term eGFR (1–3 months) (mL/min/1.73 m²)	super-selective	2	4.79 [-57.88; 67.47]	0.509	4.79 [-57.88; 67.47]	0.509	0.0%	Q=3.13, df=2, p=0.210
Transfusion (OR)	selective	3	0.80 [0.02; 26.43]	0.807	0.80 [0.02; 26.43]	0.807	0.0%	Q=1.69, df=3, p=0.640
Transfusion (OR)	super-selective	2	1.32 [0.00; 1979690.34]	0.846	1.32 [0.00; 1979690.34]	0.846	0.0%	Q=1.69, df=3, p=0.640
Overall complications (OR)	selective	4	0.45 [0.10; 1.94]	0.179	0.42 [0.12; 1.53]	0.122	19.8%	Q=3.24, df=4, p=0.519

Overall complications (OR)	super-selective	2	1.46 [0.00; 5046.79]	0.662	1.46 [0.00; 5046.79]	0.662	0.0%	Q=3.24, df=4, p=0.519
Major complications (Clavien ≥III) (OR)	selective	3	0.37 [0.02; 6.14]	0.268	0.37 [0.02; 6.14]	0.268	0.0%	Q=1.01, df=4, p=0.909
Major complications (Clavien ≥III) (OR)	super-selective	2	0.62 [0.00; 14057442.85]	0.783	0.62 [0.00; 14057442.85]	0.783	0.0%	Q=1.01, df=4, p=0.909
PSM (OR)	selective	3	0.69 [0.04; 12.17]	0.632	0.69 [0.04; 12.17]	0.632	0.0%	Q=1.34, df=4, p=0.854
PSM (OR)	super-selective	2	0.72 [0.00; 1076785.31]	0.816	0.72 [0.00; 1076785.31]	0.816	0.0%	Q=1.34, df=4, p=0.854
PSM (OR)	mixed	2	1.32 [0.00; 570.79]	0.663	1.32 [0.00; 570.79]	0.663	0.0%	Q=1.34, df=4, p=0.854

Supplementary Table S8. Subgroup analyses by surgical approach (DL+HK and REML+HK).

Outcome	Subgroup	k	Effect (DL+HK)	p (DL+HK)	Effect (REML+HK)	p (REML+HK)	I² (DL)	Test for interaction
WIT (min)	transperitoneal	5	-1.61 [-4.19; 0.96]	0.156	-1.61 [-4.19; 0.96]	0.156	0.0%	Q=0.25, df=1, p=0.616
WIT (min)	mixed	3	-1.11 [-6.26; 4.04]	0.451	-0.93 [-5.24; 3.38]	0.451	20.3%	Q=0.25, df=1, p=0.616
OT (min)	transperitoneal	6	-13.96 [-37.71; 9.79]	0.191	-24.08 [-191.82; 143.67]	0.727	66.3%	Q=7.78, df=1, p=0.005
OT (min)	mixed	2	11.20 [-422.90; 445.31]	0.798	11.48 [-142.39; 165.36]	0.517	87.4%	Q=7.78, df=1, p=0.005
EBL (mL)	transperitoneal	7	-37.01 [-148.84; 74.82]	0.449	-109.10 [-3042.50; 2824.30]	0.930	72.0%	Q=21.61, df=1, p=0.000
EBL (mL)	mixed	3	-13.14 [-79.15; 52.87]	0.482	-13.14 [-79.15; 52.87]	0.482	0.0%	Q=21.61, df=1, p=0.000
LOS (days)	transperitoneal	5	-0.15 [-0.90; 0.60]	0.616	-0.11 [-0.49; 0.27]	0.472	55.8%	Q=0.23, df=1, p=0.629
LOS (days)	mixed	2	-0.28 [-4.48; 3.92]	0.552	-0.26 [-3.78; 3.26]	0.521	28.0%	Q=0.23, df=1, p=0.629

eGFR at 1 month (mL/min/1.73 m²)	transperitoneal	2	3.27 [-62.32; 68.87]	0.640	3.27 [-62.32; 68.87]	0.640	0.0%	Q=0.28, df=1, p=0.597
eGFR at 3 months (mL/min/1.73 m²)	transperitoneal	2	9.46 [-53.46; 72.39]	0.307	9.46 [-53.46; 72.39]	0.307	0.0%	Q=2.90, df=1, p=0.089
Short-term eGFR (1–3 months) (mL/min/1.73 m²)	transperitoneal	3	7.08 [-8.12; 22.29]	0.183	7.08 [-8.12; 22.29]	0.183	0.0%	Q=1.18, df=1, p=0.277
Short-term eGFR (1–3 months) (mL/min/1.73 m²)	mixed	2	1.71 [-63.13; 66.54]	0.795	1.27 [-49.90; 52.43]	0.806	35.5%	Q=1.18, df=1, p=0.277
Transfusion (OR)	transperitoneal	6	0.65 [0.13; 3.14]	0.515	0.65 [0.13; 3.14]	0.515	0.0%	Q=0.11, df=1, p=0.744
Overall complications (OR)	transperitoneal	7	0.79 [0.41; 1.52]	0.413	0.79 [0.41; 1.52]	0.413	0.0%	Q=0.59, df=1, p=0.442
Overall complications (OR)	mixed	2	0.50 [0.00; 474.78]	0.424	0.52 [0.00; 246.88]	0.404	16.4%	Q=0.59, df=1, p=0.442
Major complications (Clavien ≥III) (OR)	transperitoneal	6	0.52 [0.13; 2.08]	0.281	0.52 [0.13; 2.08]	0.281	0.0%	Q=0.12, df=1, p=0.727
Major complications	mixed	2	0.76 [0.00; 80100.04]	0.811	0.76 [0.00; 80100.04]	0.811	0.0%	Q=0.12, df=1, p=0.727

(Clavien ≥III) (OR)								
PSM (OR)	transperitoneal	6	0.67 [0.19; 2.35]	0.446	0.67 [0.19; 2.35]	0.446	0.0%	Q=0.77, df=1, p=0.379
PSM (OR)	mixed	3	1.20 [0.17; 8.57]	0.727	1.20 [0.17; 8.57]	0.727	0.0%	Q=0.77, df=1, p=0.379

Supplementary Table S9. Subgroup analyses by tumor complexity (DL+HK and REML+HK).

Outcome	Subgroup	k	Effect (DL+HK)	p (DL+HK)	Effect (REML+HK)	p (REML+HK)	I^2 (DL)	Test for interaction
WIT (min)	low-intermediate	2	-1.92 [-19.29; 15.45]	0.394	-1.92 [-19.29; 15.45]	0.394	0.0%	Q=0.27, df=1, p=0.601
WIT (min)	mixed	6	-1.10 [-3.11; 0.92]	0.221	-1.10 [-3.11; 0.92]	0.221	0.0%	Q=0.27, df=1, p=0.601
OT (min)	mixed	7	-6.40 [-35.29; 22.50]	0.608	-22.88 [-264.77; 219.02]	0.825	78.4%	Q=2.85, df=1, p=0.091
EBL (mL)	low-intermediate	2	-9.98 [-511.09; 491.13]	0.842	-9.98 [-511.09; 491.13]	0.842	0.0%	Q=2.18, df=1, p=0.140
EBL (mL)	mixed	8	-38.97 [-122.50; 44.55]	0.306	-70.22 [-1976.63; 1836.18]	0.933	83.1%	Q=2.18, df=1, p=0.140
LOS (days)	low-intermediate	2	5.97 [-84.40; 96.35]	0.555	-0.27 [-4.46; 3.93]	0.567	87.8%	Q=0.17, df=1, p=0.676
LOS (days)	mixed	5	-0.12 [-0.49; 0.25]	0.424	-0.12 [-0.49; 0.25]	0.424	0.0%	Q=0.17, df=1, p=0.676
eGFR at discharge	mixed	2	7.29 [-45.38; 59.95]	0.329	7.39 [-43.38; 58.16]	0.316	4.3%	Q=0.02, df=1, p=0.887

(mL/min/1.73 m²)								
eGFR at 1 month (mL/min/1.73 m²)	mixed	2	5.36 [-60.28; 71.00]	0.488	5.36 [-60.28; 71.00]	0.488	0.0%	Q=0.02, df=1, p=0.902
eGFR at 3 months (mL/min/1.73 m²)	mixed	2	3.44 [-73.81; 80.69]	0.672	3.48 [-42.24; 49.20]	0.511	65.0%	Q=0.05, df=1, p=0.829
Short-term eGFR (1–3 months) (mL/min/1.73 m²)	mixed	4	4.10 [-5.80; 13.99]	0.279	4.09 [-13.20; 21.39]	0.506	8.5%	Q=0.13, df=1, p=0.722
Transfusion (OR)	low-intermediate	2	0.75 [0.00; 1024384.05]	0.837	0.75 [0.00; 1024384.05]	0.837	0.0%	Q=0.06, df=1, p=0.810
Transfusion (OR)	mixed	5	0.55 [0.10; 3.04]	0.387	0.55 [0.10; 3.04]	0.387	0.0%	Q=0.06, df=1, p=0.810
Overall complications (OR)	low-intermediate	2	0.54 [0.00; 23583.96]	0.595	0.49 [0.00; 198.88]	0.369	67.4%	Q=0.90, df=1, p=0.341
Overall complications (OR)	mixed	7	0.81 [0.42; 1.56]	0.469	0.81 [0.42; 1.56]	0.469	0.0%	Q=0.90, df=1, p=0.341
Major complications (Clavien ≥III) (OR)	low-intermediate	2	0.31 [0.00; 5375.41]	0.369	0.31 [0.00; 5375.41]	0.369	0.0%	Q=1.02, df=1, p=0.313

Major complications (Clavien ≥III) (OR)	mixed	6	0.82 [0.18; 3.62]	0.741	0.82 [0.18; 3.62]	0.741	0.0%	Q=1.02, df=1, p=0.313
PSM (OR)	low-intermediate	2	0.79 [0.00; 5404.05]	0.790	0.79 [0.00; 5404.05]	0.790	0.0%	Q=0.06, df=1, p=0.808
PSM (OR)	mixed	7	0.96 [0.38; 2.43]	0.909	0.96 [0.38; 2.43]	0.909	0.0%	Q=0.06, df=1, p=0.808

Supplementary Figure S1. Risk of bias (ROBINS-I) for non-RCTs.

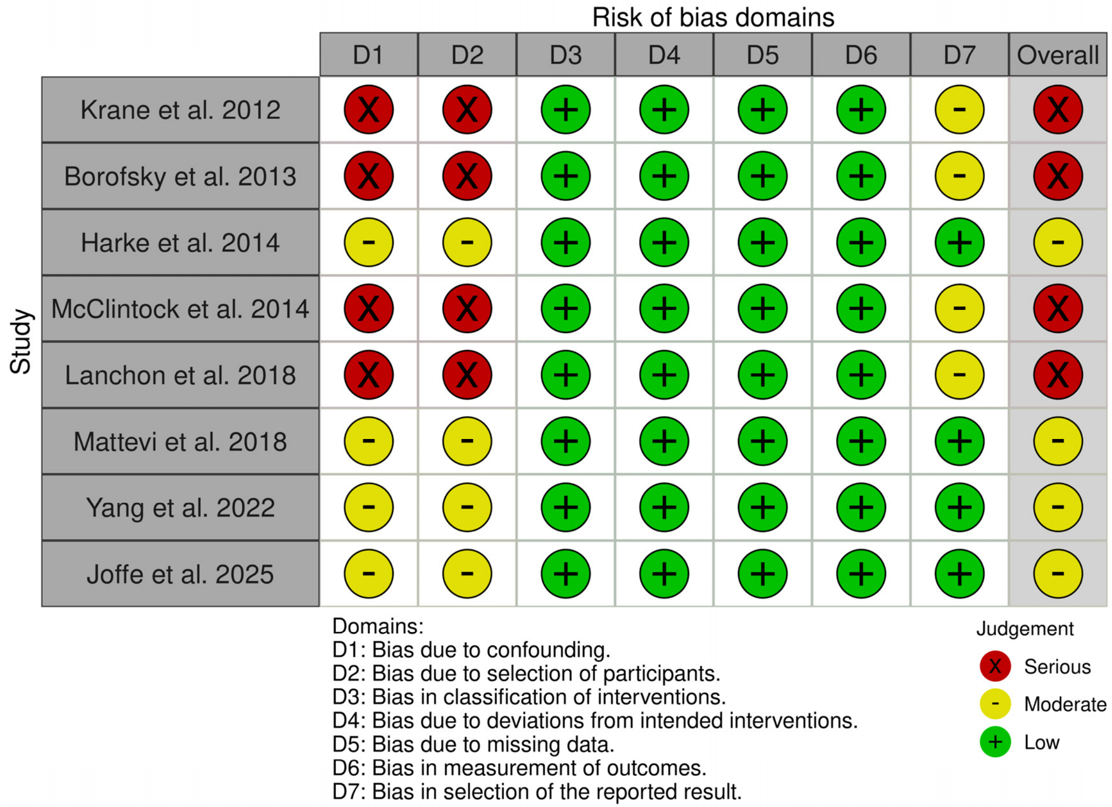

Supplementary Figure S2. Risk of bias (RoB 2) for RCTs.

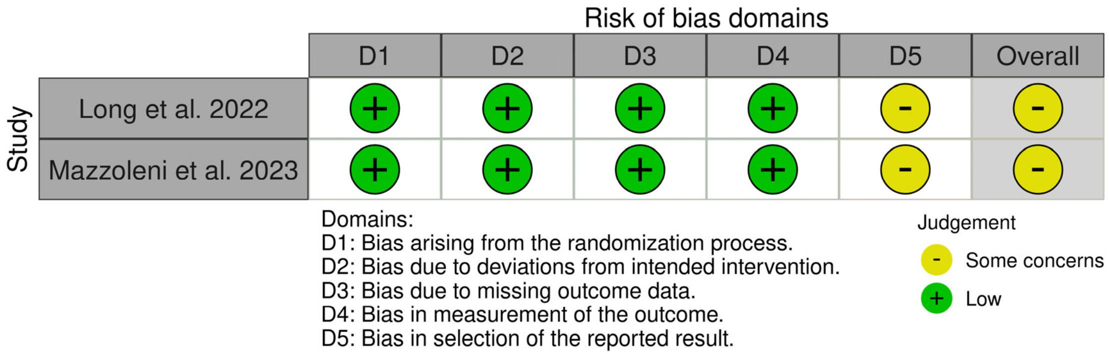

Supplement: Supplementary file 1 [file medicina-61-01735-s001.zip › medicina-3827355-supplementary.pdf]
